# A concept for multi-winner tenders for medicinal products with balancing between efficient prices, long-term competition and sustainability of supply

**DOI:** 10.3389/fmed.2023.1282698

**Published:** 2023-10-30

**Authors:** Gergely Németh, Mánuel László Mágó, Zoltán Kaló, Judit Lám, Tamás Balogh, Valentin Brodszky

**Affiliations:** ^1^Data-Driven Health Division of National Laboratory for Health Security, Health Services Management Training Centre, Semmelweis University, Budapest, Hungary; ^2^Department of Reimbursement, National Institute of Health Insurance Fund Management, Budapest, Hungary; ^3^Institute of Economics, Corvinus University of Budapest, Budapest, Hungary; ^4^Center for Health Technology Assessment, Semmelweis University, Budapest, Hungary; ^5^Department of Health Policy, Corvinus University of Budapest, Budapest, Hungary

**Keywords:** multi-winner tender, price efficiency, shortage prevention, supply guarantee, sales guarantee, equity in access

## Abstract

Achieving price efficiency via tenders, the sustainability of competition, and the prevention of shortages are hot topics in the debates about shaping the pharmaceutical markets. Single-winner tenders receive growing criticism for concentrating on achieving low prices at the expense of the long-term maintenance of a competitive pharmaceutical industry, the security of continuous supply, and disregarding the therapeutic needs of patient populations with specific conditions. This paper aims at drafting a concept to assist the design of multi-winner tenders for medicinal products with a focus on supply and sales guarantees, price efficiency, and equity in access. The concept shall be generally applicable to all kinds of medicinal products including generics, biosimilars, and on-patent products in the out- and in-patient sector. Principles for multi-winner tenders for medicinal products are set and a number of delimitations are made in order to get rid of factors that prevent clairvoyance amid the various pricing and reimbursement systems when designing a concept. The steps to plan and implement a multi-winner tendering procedure are drafted on the basis of the defined principles. The tender should consist of planning, bidding, preparation, sales, and evaluation phases. Pharmaceutical companies shall make bids with price and quantity pairs, which shall be ranked by prices and if applicable then taking into account other factors. The tenderer shall predefine market shares to the various places of the ranking. A double ceiling shall be applicable for the sales of the winners: their sales must not exceed their quantity offer and the predefined market share applicable to their place in the ranking. The implementation of the concept will require the careful adjustment of the tender conditions to the specificities of the pharmaceutical market concerned on the one hand and to the local pricing and reimbursement system on the other hand.

## 1. Introduction

Ensuring effective prices for pharmaceuticals is a ubiquitous effort of national governments. A wide range of pricing policy tools is applied worldwide (1) with very different design and implementation patterns (2). Tendering is one of these pricing tools widely used for enhancing competition (1, 3). The design and application of tenders vary across (2, 4) and even within countries (5). Tenders are generally used in the hospital sector for purchasing pharmaceuticals in Europe (6), and primarily single-winner tenders are in place (2).

In the outpatient sector, the usage of tendering is seemingly less widespread. Most European countries refrained from its introduction and introduced an internal reference pricing system instead (e.g., Belgium, Hungary, and Portugal (2)). From a theoretic economic perspective, these internal reference pricing systems could be considered multi-winner tendering-like systems because the system designs do not exclude sales of multiple companies on the market, and certain privileges are assigned to the best-priced products.

Tendering for biosimilars is general in the EU; most countries introduced tenders, at least for certain biosimilar areas (7). Tendering is also applied to on-patent medicinal products.^1^ Though it would be challenging to attribute price level to a single factor within the complex pharmaceutical pricing landscape, it still seems that countries with single-winner generic tendering systems (e.g., the Netherlands, Slovakia (2, 8)) or countries with single-winner tendering like systems (e.g. Sweden (9), Denmark (10)) in the out-patient sector are usually among countries with the lowest-priced generics at least in the European Union in the annual price analyses of the Swedish Dental and Pharmaceutical Benefits Agency (11). Belgium launched a tendering system in 2007 but withdrew it the next year because one of the winning companies had no capacity to procure (12). Vogler et al. in their analysis of the Belgian, Danish, and Dutch off-patent tendering system concluded that “policymakers should consider the establishment of a robust legal and organisational framework, a strategic design of the tendering policy, the development of strategies to avoid or at least address shortages, appropriate stakeholder management and demand-side policies to promote generic uptake. After the introduction, it is suggested to monitor the performance of the tendering policy and adapt its design if needed” (12).

Single-winner tenders receive criticism, especially from the pharmaceutical industry (2). There are general concerns about the impact of tendering on patients' access to medicines. These concerns can be based on four main arguments. First, the long-term effect of single-winner tenders is unclear (13), as it may lead to decreased competition (14) in the long run because the renewal of tenders may be associated with significant transaction costs both for the manufacturers and health care system (i.e., switching all patients on chronic maintenance therapies) if the single-winner changes. The case of pemetrexed in Hungary seems to support this assumption. While the first years of single-winner tenders resulted in very low prices, other manufacturers withdrew from the Hungarian market. Years later the single manufacturer raised the price of pemetrexed after having several issues with the continuous supply. Second, single-winner tenders carry an increased risk of shortage of supply (12, 15). Third, single-winner tenders might be more associated with increased incentives for corruption in countries with limited tradition for objective and verifiable selection criteria in tendering and independent audit of tender practices (16). Finally, if single-winner tenders are applied for medicines that are not fully equivalent in terms of bioavailability, real-world effectiveness, and safety, there is no guarantee that the selected medicine is the optimal choice for all patients, therefore, single-winner tenders cannot support equity in patient access according to medical needs. This concern was raised already for the therapeutic reference grouping (17) and is relevant for tenders with different pharmaceutical products as well (18).

Price levels of the off-patent markets in those countries where internal reference pricing is in place, are close to average among European countries with quite different successes in assuring maximum price efficiency. The price efficiency in Finland, where the internal reference pricing system (19) is the main policy tool for pricing of generics, is close to the best-performing tendering or tendering-like systems in Sweden, Denmark (11). The performance of the Hungarian, German, or Portuguese internal reference pricing system seems significantly worse, still, they have quite good positions in the European ranking (2, 11). In contrast the Czech, Greek, and Italian internal reference pricing systems result in fairly high prices (2, 11). This experience raises the concern that these schemes are not efficient in the economic sense, even beyond the measure that could be expected in a multi-winner environment.

Internal reference pricing systems are complex and very diverse in their design. The numerous factors that impact the system's outcome (like the period of recalculating the prices, co-payment schemes, substitution regulation, demand-side measures, or the privileges assigned to the lowest-priced products and the additional policy tools applied e.g. external reference pricing) make the comparison and assessment difficult. The general assumption in internal reference pricing systems is that the presence of several suppliers mitigates the risk of a shortage of supply. Internal reference pricing systems do not have built-in mechanisms to guarantee the market supply.

Multi-winner tenders for pharmaceuticals seem challenging to plan and implement compared to single-winner tenders, as several factors are recommended to be considered (20). Multi-winner tenders are applied in other industrial branches with the theoretical support of auction, tender, and game theory (e.g., electricity or mobile network tenders). Throughout less widespread, multi-winner tenders are in place in a few countries in Europe (e.g. Germany, Spain, Austria, Greece, Hungary, Italy, Portugal, United Kingdom (2)), still there is a lack of a general framework for designing these tenders.

In this paper, an attempt will be made to design a tendering concept for medicinal products that could be generally applied to all kinds of pharmaceuticals considering the general specificities of the pharmaceutical markets and addressing the most important challenges in designing multi-winner tenders. The concept could be applied in all kinds of settings where a multi-winner tender is needed: in an aggregated national-level procurement, on the district, cantonal, provincial, or regional level, or in a single hospital or health facility.

## 2. Conceptualising multi-winner tenders

Although in the literature several principles for designing tenders for medicinal products are recommended to be taken into account (4, 20, 21), this paper will concentrate on the following five principles:

- multiple winners (having a limited number of suppliers in accordance with the market, product, and country characteristics), thus avoiding the risk of the evolution of a monopoly in the long run,
- supply guarantees to help the prevention of shortages of supply,
- sales guarantees for the winners, which is a prerequisite for efficiency of the tender from the auction theory perspective (22–25),
- assurance of price efficiency at its limits in the multi-winner environment from a theoretical economic perspective,
- guarantee of equity in access to therapy for patients with special needs.

Additionally, the following expectations regarding the tendering concept are defined.

The tendering concept should be generally applicable to the health care systems of different countries. However, feasibility and stability of policy implementation should be ensured by considering local settings, particular health system, current and future institutional structures, and the availability of human and financial resources. For the general applicability of the tendering concept, some limitations must be stated.

The tendering concept will not deal with the eligibility of products participating in the tender. Nevertheless, two conditions must be clarified in this regard: first, new products which were not winners of the tender cannot get reimbursement during the sales period; second, the dispensing of products with reimbursement which are not among the winners must be prohibited.

The tendering concept will not deal with the pricing of the products participating in the tender. In particular, how the list prices of the products participating in the tender are set and the relation between the list prices and the prices offered in the tender. A part of the literature (27, 28) discusses tenders with other criteria besides the price.

The tendering concept will not deal with interactions with remuneration schemes for wholesalers and pharmacies. In many countries, the remuneration of pharmaceutical wholesalers and pharmacies is regulated by law. Regulation influences the behaviour of the mentioned actors in the supply chain; thus, it affects the demand for medicinal products. The concept assumes that the remuneration scheme for wholesalers and pharmacies does not contain incentives that undermine the tendering concept's application and effectiveness.

The tendering concept will not deal with patient co-payment schemes. Patient co-payment schemes have a significant impact on the demand for medicinal products. Patient co-payment schemes differ significantly among countries (e.g., fixed co-payment and price percentage-based co-payment). Furthermore, co-payment schemes can be reasonably different even within a given country (e.g., exemptions from co-payment and mixture of fixed and percentage-based co-payments, caps in co-payment, etc.), and in many countries, there is no co-payment at all for at least certain medicinal products (hospital only medicines particularly). The concept assumes that the co-payment scheme, if applicable, does not contain incentives that undermine the application and effectiveness of the tendering concept.

The tendering concept will not deal with legal aspects, nevertheless, the tender design must adhere to the applicable law on public procurement (20). The tendering concept will not deal with the organisational aspects of the design and running of tenders, though these are prerequisites for successful tenders (29).

The concept primarily considers the price as the ranking criterion among the bids, however it allows that other factors (e.g., local manufacturer, quality of products, prolonged dosage form, etc.) be considered for the ranking. In such a case, these factors shall be clearly defined and communicated in the planning phase in line with recommendations on the application of multiple criteria for the selection of winners (30).

In Section 2.1 the tendering concept's theoretical principles will be discussed and then explained how the idea could work.

### 2.1. Basic considerations

#### 2.1.1. Multiple winners

A starting point is the assumption that one pharmaceutical company would be able to supply the whole market. The consequence of this consideration is that some sales quotas (percentages of the market shares) shall be introduced to allow the presence of multiple suppliers.

The second point is that the demand is difficult to be predicted (20, 31), as many factors influence the actual demand. Consequently, sales quotas shall be based on market shares and not on amounts of units sold.

Some of the key elements of the tendering procedure introduced in this paper have similar parallels in the literature regarding renewable electricity auctions. Specifically, based on some theoretic economic considerations the usually better auction practices for the renewable energy market includes volume disclosure, price ceilings, penalties, a schedule for auctions, a streamline of administrative procedures, and provision of information to potential participants (32), although there may not be an exact blueprint for a good auction design (33).

#### 2.1.2. Supply guarantees

Probably the most critical problem with multiple-winner tenders is to determine whose responsibility the supply of the market is. Can any supplier be blamed if there is insufficient supply, bearing in mind that the tenderer cannot predict the exact demand? The suggested solution is that pharmaceutical companies shall be requested to offer the quantity they can deliver, and this offer shall be binding. Each pharmaceutical company shall be responsible for delivering the offered amount only, independently from the behaviour of other pharmaceutical companies or the changes in the demand compared to predictions. Consequently, pharmaceutical companies must offer price and quantity pairs, not only prices.

The other point in terms of supply guarantees is the prevention of shortages. The tendering system itself shall contain measures that contribute to preventing shortages.

The most crucial measure shall be that the quantity offers shall be published together with the prices and rankings. This information will allow participating pharmaceutical companies to adjust their production and delivery to meet the demand according to their ranking and the forecasted delivery of other pharmaceutical companies.

Additionally, the following factors shall contribute to guaranteeing market supply:

- pharmaceutical companies shall be incentivised to deliver the offered quantity, if necessary, by imposing sanctions if they fail in the delivery,
- pharmaceutical companies shall have enough time between bidding and the start of the sales to manufacture and deliver the offered quantity,
- above the predefined market shares, pharmaceutical companies shall be allowed to supply the market up to the cumulative market shares of the higher-ranked bidders, and
- if the winners of the tender are unable to supply the market, then other pharmaceutical companies shall be allowed to supply the market with the prices they had offered.

#### 2.1.3. Market share guarantees

Auction theory suggests that an auction can be efficient if the conditions of the auction incentivise the bidders to tell the truth about their valuations and the most favorable bidders are rewarded (22–25). A price-quantity bidding tender can be considered similar to an auction as participating firms compete in their prices to win market shares. The main difference is that there can be multiple winners, as many firms may supply a given market. The question is then how truth-telling can be incentivised in the quantity bids and how prices can be lowered generally. Regarding the prices, the incentive to compete (lower prices) is that the lowest price bids shall be ranked higher, and the reward is that the highly ranked products shall have priority for sales. Regarding the quantities, the incentive for telling the truth is that - as a rule of thumb - selling less or more than offered shall be sanctioned, with some exceptions explained later. The reward for the quantities is that bidders get sales guarantees for the offered amount up to the market shares predefined for the places in the ranking (predefined market shares). The higher ranking shall be associated with higher market shares in order to prevent perverse bidding strategies.

In case of violating the tender rules, innocent pharmaceutical companies, who suffered losses, shall be compensated from the sanctions imposed.

#### 2.1.4. Price efficiency

As said earlier, from the theoretical economic perspective (25) a single-winner tender would result in efficient (lowest possible) prices (based on the assumption that each pharmaceutical company could supply the whole market), at least in the short run, if the price is the only or at least dominant selection criteria. The multi-winner tender concept assumes that the price paid above the short-run equilibrium price in a multi-winner environment balances the price premium that would have to be paid to a monopoly (single) supplier in the long run. The equilibrium point is unknown and may differ between the various pharmaceutical markets, which vary in size, market entry costs, etc. In this paper, no attempt will be made to define the equilibrium point. Still, a framework will be set up in which the equilibrium price could be reached empirically by specifying the number of winners and the length of the tender period. Consequently, the concept shall be flexible in determining the number of winners and the length of the tender period. A framework for multi-winner tenders for spectrum auctions is introduced in Wu et al. (26) and some similar considerations are discussed.

#### 2.1.5. Equity in access

In an ideal situation, the competing products in the tender should be perfect substitutes. This is rarely the case: there are differences in the excipients, the dosage forms, or even in the active substances. Though these differences may not result in different treatment outcomes for most of the patients, some patients may be intolerant (34) or allergic to certain excipients (e.g. lactose); the same dosage form may not be suitable for certain patients for the administration of the medicine (e.g., too large tablet for patients with difficulties in swallowing instead of an oral liquid form), some patients may have special clinical conditions which significantly influence the bioavailability or elimination of active substances (e.g., renal failure).

While single-winner tenders carry the risk in themselves that the tender winner product may not be able to satisfy the therapeutic need of all patients (18), the greater variety of the available products in a multi-winner tender increases the probability of finding the appropriate medicinal product for patients with special needs. Yet a multi-winner tender itself cannot guarantee equity in access for all patients, which necessitates a plan for patients with special needs.

### 2.2. The proposed tendering procedure

In order to achieve the defined objectives—keeping the conditions listed above in mind—the following tendering procedure is proposed. Pharmaceutical companies shall make bids with price and quantity pairs which shall be ranked by prices and if applicable then taking into account other factors. The tenderer shall predefine market shares to the various places of the ranking (predefined market shares) and define the number of winners at the same time. As a general rule a double ceiling shall be applicable for the sales of the winners: their sales must not exceed their quantity offer and the predefined market share applicable to their place in the ranking.

The proposed tender comprises five phases: planning, bidding, preparation, sales, and evaluation. In the case of consecutive tenders, the phases recur and can overlap. The sales phase cannot overlap with another one for the same market. The phases are described in the following sections. The concept does not give guidance on the lengths of the phases. They should be determined by the concrete tenderer considering the characteristics of the market concerned and the local circumstances. The concept does not give guidance on how many winners there should be.

#### 2.2.1. Phase 1—Planning

The main goal of this phase is to set up and communicate all rules regarding the complete tender.

The tenderer shall set at least the following parameters and documents:

- the market itself, namely the scope of products that are eligible to be included in the tender (e.g., one active substance, certain pharmaceutical forms of an active substance, combined products, different active substances, etc.),
- the units in which quantity bids shall be made and the market shares shall be measured (e.g., mg of active substances, number of pills, number of patients treated, etc.),
- the units for which the price bids shall be made (e.g., price per mg, per ml, per tablet, the average price of a tablet, per successfully treated patient, etc.),
- the length of the planning, bidding, preparation, sales, and evaluation phases,
- the predefined market shares (and consequently the number of guaranteed winners),
- selection and decision criteria for the tender winners and algorithm for aggregating results in case of multiple criteria,
- commitments for minimum quantities purchases, if applicable,
- minimum quantities for bids, if applicable,
- the way of monitoring the market shares,
- the tolerance level for deviation from the predefined market shares,
- the tiebreaking rules,
- the mechanism of how the case of a shortage can be declared,
- the general rules of evaluation and dispute-setting mechanisms,
- the sanctioning and compensation mechanisms,
- the conditions of an unsuccessful tender (e.g., minimum quantity offers, too high prices, etc.),
- a plan to support compliance with the tender rules (compliance plan), which must include a measure for the tender winners, prescribing doctors, dispensing pharmacists, and patients to support the compliance (e.g., prescriptions and dispensing must be aligned with the predefined market shares, switches must be managed, etc.),
- a plan how equity in therapy access for patients with special needs (bioavailability, etc.) will be guaranteed.

The predefined market shares serve as the base plan for dividing the market in the sales phase. These market shares are one of the essential decision variables of the tenderer, as their value decides the number of possible winners the tender can have. It is important to note that the proposal has the flexibility to cover the case of single-winner tenders as well by setting the maximum market shares for the lowest price bidder to 100%, however in such a case most of the shortcomings of single winner tenders discussed earlier have to be reckoned with. Producers may be subject to sanctions if they do not comply with these market shares. The tolerance level can dampen the strictness of the predefined rules.

The units in which the market shares are defined are the other key parameter of a tender. The definition of units (e.g., mg; international unit, tablets, number of patients treated, number of naïve patients treated, etc.) must be in line with the pharmaceutical (e.g., solid oral forms, or all oral products, etc.), price (is flat pricing or is per mg price applied), and therapeutical characteristics (e.g., once daily tablet, starting and maintenance dose of therapy, etc.) of the products included into the tender. The options for defining the unit would allow the flexible usage of the tendering concept, meaning that it could be used for generics, biosimilars, or even competing on-patent products. The price offers shall be in line with the units (e.g., price per mg or average price per mg, price per international unit, price per tablet, price per treatment).

In the case of consecutive tenders, the first planning would require more effort, if the conditions of the tender are set optimal, then the planning phase could be a routine exercise.

#### 2.2.2. Phase 2—Bidding

In this phase, pharmaceutical companies shall submit their bids according to the pre-determined rules of the tender.

The bidding phase starts with the official posting of the tender and ends with the pre-determined closure of the bidding process. During the bidding phase, participating pharmaceutical companies place blind bids by submitting price-quantity pairs. The individual bids of the pharmaceutical companies at the time of the bidding decision are confidential private information. Still, once the bidding process is finished, the tenderer shall publish all valid price-quantity bids. The bidding procedure shall ensure that pharmaceutical companies make their decisions solely based on the public information available and based on their private information about their production capacities and intentions.

Pharmaceutical companies shall be ranked in a way that the cheapest (the one with the lowest price bid) comes first, then the rest in ascending order based on the price bids. In case of ties, pharmaceutical companies with the exact same prices are initially ranked together and the given tiebreaking rules determine their final order. Once the order of the pharmaceutical companies is determined, they are matched with the predefined market shares. The number one spot goes to the cheapest, the number two spot to the second cheapest and so on. In the case of ties, several tiebreaking rules can be applied. One possibility could be to split the summed-up market share of the tied spots evenly between the spots. Another option is to split them proportionally to the quantity bids of the tied pharmaceutical companies and let the ones with the higher quantity bid take the earlier spot. Suppose the bids of some pharmaceutical companies are completely the same (both in their prices and their quantities). In that case, a simple randomisation (a coin-toss of some sort) can also decide which pharmaceutical companies get the more beneficial earlier spots.

The bids (including both prices and quantities) must be published after the deadline for the bids, which marks the end of the bidding phase. By knowing the predefined market shares, the ranking, and the offered quantities of the competitors and making forecasts on the market size evolution based on the previously mentioned information, pharmaceutical companies shall adjust their production and delivery in the preparation phase.

Commitment to the bids is a crucial element of the tender. The submitted quantity bids are interpreted as amounts the pharmaceutical companies could be expected to deliver if needed. As such, the tenderer might sanction those pharmaceutical companies who are over- or underachieving their bids in terms of quantities sold. The proposed sanctioning mechanism is described in more detail during the evaluation phase. It shall be impossible to change prices until the next bidding phase; pharmaceutical companies have to respect market shares and comply with their offered quantities.

#### 2.2.3. Phase 3—Preparation

In this phase, pharmaceutical companies shall prepare the delivery of the proposed amounts adjusted to the expected sales taking into account the outcome of the bidding process (including their own ranking and the bids of others), the predefined market shares, and their market dynamics forecasts. The length of the preparation phase shall enable pharmaceutical companies to manufacture and deliver the submitted quantities. The optimized length of the time period shall reduce the probability of supply shortages.

#### 2.2.4. Phase 4—Sales

In this phase, pharmaceutical companies shall deliver the promised quantity of their products adjusted to the predefined market shares and market dynamics, which could be overruled in case of a shortage (see detailed description below). The sales phase starts after the end of the preparation phase and lasts for a pre-determined period of time. The concept does not deal with the optimisation of the length of the sales period. Several factors may influence the optimal length of the sales period, like the number of pharmaceutical companies, characteristics of the therapy, transaction costs (35), etc.

During the sales phase, the tenderer and/or the pharmaceutical companies shall monitor sales to keep the predefined market shares and avoid sanctions. Sanctions (discussed later) incentivize pharmaceutical companies to stick to the predefined market shares and their quantity bids. The possibility of sanctions might incentivise the winners to cooperate during the sales period to keep the predefined market shares. The biggest challenge of the concept is to find how the market shares could be monitored during the sales phase. Ideally, the monitoring tool shall be the same as used for calculating market shares in the evaluation phase. Tracking the market shares could be easier if the procurement of the medicinal product is organised centrally (e.g., in the case of hospital-only products). In the case of a tender in the outpatient sector, sales monitoring would be more difficult. An option is a data warehouse where the winners upload their delivery to the wholesalers so the winners can predict the evolution of market shares. Another option in the EU is using the European Medicines Verification System (EMVS^2^) for this purpose. In the latter case, the pharmacy reimbursement payment should be linked to decommissioning in the EMVS. Additionally, the compliance plan can have further ideas how the predefined market shares can be kept by during the sales phase (e.g., randomised prescriptions adjusted to the predefined market shares).

In the sales period, managing potential shortages is a crucial question. Pharmaceutical companies are expected to manage the fluctuation in demand by adjusting their production and delivery up to the offered quantities. The expectation is that the offers altogether will far exceed the demand. Nevertheless, shortages might occur. The supply of the market shall have priority over the predefined market shares and quantity offers. Therefore, the case of a shortage must be publicly declared, and the conditions for the declaration shall be defined and communicated in the planning phase.

Two types of shortages shall be managed. The first type is the product level shortage, when one or more pharmaceutical companies do not deliver the offered quantity, but other pharmaceutical companies can supply the market. In such cases, the next ranked winner can take over the market supply up to its cumulative market share. The pharmaceutical company(ies) that did not deliver the necessary quantity shall be sanctioned.

The second type of shortage is the general shortage when the winners could not meet the demand on the market. A general shortage can have three reasons: 1. several pharmaceutical companies delivered less quantity than offered which led to a general shortage (these pharmaceutical companies shall be sanctioned); 2. the offered quantities (though delivered) were not enough to supply the market; or 3. there was a significant increase in the demand, which could not be met with offered quantities. In such a case, any pharmaceutical company (including outside parties) should be allowed to supply the market at their prices. The supply of the market shall have a preference over the accepted prices.

It must be noted that shortage prevention is different from the goal of the concept. Still, multi-source purchases can increase the supply reliability of pharmaceuticals and the resilience of health care systems in under special circumstances (e.g., pandemics, wars, or national economic crises in regions with significant manufacturing capacities). The enhanced predictability of sales can help pharmaceutical companies in better planning production, thus preventing shortages. Still, shortages can occur due to force majeure, health, or economic crises.

#### 2.2.5. Phase 5—Evaluation

The evaluation phase starts after the sales period and concludes with an official statement about the procedure. The goal of the evaluation is to declare if the pharmaceutical companies were compliant with their offers and the predefined rules. If the rules of the tender were violated, then corrective measures shall be taken: sanctions and potential compensations shall be imposed. Pharmaceutical companies shall have the opportunity to appeal against the decisions of the tenderer on sanctions and compensations. The evaluation phase and the whole tender procedure shall be terminated when all—if any—disputes are settled.

During the evaluation, the following comparisons and checks shall be made to state if any violation of the offers or the tender conditions occurred:

1. Comparison of the quantities sold to the offered quantities.
2. Comparison of the actual market shares to the predefined individual market shares.
3. Comparison of the actual market shares to the predefined residual cumulative market shares.
4. Checking if there was a shortage on the market.

## 3. Sanctioning scheme

As mentioned earlier, the sanctioning scheme aims to incentivize pharmaceutical companies to comply with their price and quantity offers and the predefined rules of the tender procedure. The sanctioning scheme must not be abused to reduce the purchasing cost of medicinal products because it may discourage pharmaceutical companies from participating in tenders.

In the following paragraphs, the principles of the sanctioning scheme will be described, particularly when sanctions shall be imposed and how the quantity basis for the sanction and the amount of the sanction shall be defined. A non-financial measure other than monetary sanctions might also be applied. The tenderer can sanction (continuous) noncompliance with a ban from tenders for a given period or even indefinitely.

### 3.1. Principles

The following principles are suggested for the sanction scheme:

- pharmaceutical companies compliant with their quantity offer and the conditions of the tender shall not be sanctioned under any condition,
- sanctions shall exclusively depend on the behaviour of the pharmaceutical company concerned and must be independent of the behaviour of the competitors,
- the most minor difference between the units sold and the would-be number of units sold by compliant behaviour shall be the basis for the sanction,
- the sanction shall be high enough to disincentivize non-compliant behaviour but must not be too high to disincentivize pharmaceutical companies to participate in tenders,
- the tenderer shall determine sanctions in advance.

#### 3.1.1. When shall sanctions be imposed?

1. If a pharmaceutical company exceeded both its individual and cumulative market shares and there was no general shortage on the market.
2. If a pharmaceutical company sold less than its offered quantity, neither its individual nor its cumulative market shares were reached (irrespective of a shortage).
3. If a pharmaceutical company sold more than its offered quantity and there was no shortage.

#### 3.1.2. How shall the quantity basis for the sanction be determined?

To determine the amount of the sanction, first, the most minor change in the quantity sold by the pharmaceutical company has to be determined that would have resulted in compliant behaviour. Second, the exact fine is calculated based on a given rule.

1. Determination of the quantity deviation from the compliant behaviour.

It is possible to find the closest compliant outcome for every non-compliant behaviour given the bids of the participants and the actual outcomes. Under any non-compliant scenario, a hypothetical outcome can be determined requiring the smallest possible deviation (in quantity terms) to the closest compliant behaviour from the actual outcome. So, for example, if a pharmaceutical company is sanctioned because it sold more than what it offered (and there was no shortage), the most minor decrease has to be found in its sold units so that it would not be sanctioned anymore.

Notably, a tolerance can be set for violations of the offered quantities and the market share requirements. So, for example, if a pharmaceutical company sells an amount in a 5% neighbourhood of its compliant behaviour, it counts as meeting that requirement. The tolerance rate shall only be applied if all winners fall inside the tolerance rate.

2. Calculating the amount of the sanction

Three options are proposed for calculating the amount of the sanction based on the previously determined quantity deviance. Further discussion on these issues is necessary to specify in which case the specific options shall be used.

1: Fine is determined based on the price of the product of the given pharmaceutical company. Under this rule, if a pharmaceutical company is sanctioned for a given quantity, then the sanction imposed is a given percent of the value of that quantity, evaluated at a price set by the pharmaceutical company. This option can be used if the cheapest product shall be sanctioned or if the difference between the products in the ranking is too little or zero.
2: Fine is determined based on the price difference between the price of the pharmaceutical company and the next higher price. Under this rule, if a pharmaceutical company is sanctioned for a given quantity, then the sanction imposed is calculated by multiplying the quantity by the difference in the prices of the pharmaceutical company and the next higher price. The last ranked winner shall not pay any sanction according to this option as it cannot be punished for any number of boxes. This rule ensures that if the tenderer incurs higher costs due to a non-compliant pharmaceutical company, the non-compliant pharmaceutical company covers this extra cost.
3: Fine is calculated based on comparing total costs in two scenarios. The full value of the actual quantities sold shall be calculated and compared to the cheapest possible way demand could be met, so that all pharmaceutical companies are compliant. The most inexpensive compliant way to supply the market might be overall more or less expensive than the realised total costs. In this idealistic scenario, the would-be income of every pharmaceutical company can be calculated. Compliant pharmaceutical companies should not pay any fines. Non-compliant pharmaceutical companies should pay the difference as a fine if their realised gain is higher than their idealistic would-be income. In contrast, if their realised income is lower, they should pay a quantity-based fine, as discussed earlier. Compliant pharmaceutical companies with realised gains lower than their idealistic would-be income might be compensated up to their would-be income.

## 4. Compensation scheme

### 4.1. Principles

The following principles are suggested for the compensation scheme:

- the total value of the compensations shall not exceed the total amount of sanctions imposed. Therefore, if no pharmaceutical companies are sanctioned, then no pharmaceutical companies can receive any compensation either (see below),
- if a pharmaceutical company is sanctioned, it cannot receive a compensation,
- the pharmaceutical companies shall individually request compensation, and the validity of their claims must be proven by themselves.

There are multiple reasons why the total amount of compensation should not exceed the total amount of sanctions imposed. First, if this principle is met (at least in the long run, over several periods), then the tenderer should not inject additional funds into the tendering system to keep it running. Second, due to the setup of the concept, pharmaceutical companies can only receive compensations for units they could have sold if some other misbehaving pharmaceutical companies were compliant with their bids and market shares. Therefore, the number of units for which pharmaceutical companies receive compensation cannot exceed the number of units for which other pharmaceutical companies receive sanctions. It is possible though that the lost income of the would-be-compensated pharmaceutical companies is higher than the income that the to-be-sanctioned pharmaceutical companies realised via their misbehaviour if cheaper products exceeded their market share). Still, compensation is not entirely meant to compensate pharmaceutical companies for their lost income, but to help non-winning, or smaller pharmaceutical companies to stay in the business and to create competition in the long run. The unsold medicinal products could be sold in the next tender. Finally, if a per-unit compensation could be larger than a per-unit sanction, then it may incentivise pharmaceutical companies to strive for compensation, even at the cost of non-compliant behaviour and sanctions.

It is possible that in case of some violations a pharmaceutical company might seem to be eligible for compensations and sanctions at the same time (e.g., if the pharmaceutical company sold less than its quantity offer, and its predefined market share - this could have two reasons: 1. the pharmaceutical company delivered less quantity than its offer, which should be sanctioned or 2. other pharmaceutical companies superseded their market shares on its expense, which loss could be compensated). However, in these cases, the burden of proof is always on the pharmaceutical company to show that it is eligible to receive compensation and should not be sanctioned.

#### 4.1.1. When might compensation be requested?

Compensation can only be paid if a pharmaceutical company sells less than its quantity bid and does not exceed its market share requirement, and it can prove that this was due to the behaviour of others.

#### 4.1.2. How shall the amount of the compensation be determined?

The amount of compensation shall be determined on the following basis:

1. Compensations can be up to the amount of sanctions imposed on those pharmaceutical companies from which the receiving pharmaceutical company can prove that they have caused losses.
2. Compensations can be calculated based on the number of boxes the pharmaceutical company could have sold if all the pharmaceutical companies were compliant. Like this, pharmaceutical companies can be compensated up to the value they could have sold in the closest compliant scenario.

## 5. Discussion and conclusion

The authors advocate for designing and implementing multi-winner tenders for pharmaceuticals instead of single winner tenders. Multi-winner tenders can contribute to the long-term competitiveness of the pharmaceutical markets and as a consequence can contribute to the maintenance of price efficiency. Additionally multi-winner tenders may mitigate the risk of shortages of supply and can better meet the therapeutic needs of patient population with special conditions. The concept addresses the challenges of introducing sales and supply guarantees in the multi-winner environment and suggests measure to further decrease of risk of shortages.

The concept is delimited from wholesaler and pharmacy remuneration, reimbursement systems, and patient co-payment schemes. These delimitations allow the general applicability of the concept within different health care systems and various pharmaceutical sectors (out-patient and hospital; off-patent and competing on patent) on the one hand but would require careful planning in the adaptation to the local conditions and specificities of the tender market on the other hand. The concept does not consider other aspects of the tender than the price; however it allows the inclusion of additional selection criteria for the winners.

The concept suggests five phases for the tender procedure. Successful tendering requires detailed and comprehensive planning considering the specificities of the affected market and the local conditions (reimbursement, co-payment schemes, treatment characteristics, characteristics of the medicinal products, etc.). The rules of the tendering and the bids (price and quantity pairs of all offers) must be published. The concept offers flexibility in determining the number of winners, and cannot determine the optimal frequency for launching tenders. The most common tender length in Europe is 13–24 months (36) and the similar duration is applied in several non-European countries (3). These parameters shall be considered during the planning phase.

Transparency and clear communication during the whole period of the tendering are essential: the tenderer and the pharmaceutical companies must cooperate to allow the achievement of the predefined market shares for the winners and help each other avoid shortages.

The compliant behaviour of the tenderer and the pharmaceutical companies with the tendering rules must be anticipated. However the planning must cover the consequences of the non-compliant behaviour of any party. Corrective measures shall be set up for the case of non-compliant behaviour: sanctions and compensations shall be imposed, if necessary, to balance wins and losses due to breaking the rules. The application of the corrective measures shall be further elaborated and adapted to concrete tenders.

Careful implementation of the concept can help enhance competition on the pharmaceutical market, while contributing to the sustainability of competition in the long run and the prevention of shortages. The experiences of tenders where the suggested mechanisms are implemented shall be gathered and evaluated to help fine-tune the concept's implementation.

## Data availability statement

The original contributions presented in the study are included in the article/supplementary material, further inquiries can be directed to the corresponding author.

## Author contributions

GN: Conceptualization, Methodology, Writing—original draft. MM: Conceptualization, Methodology, Writing—original draft. ZK: Conceptualization, Writing—review & editing. JL: Supervision, Writing—review & editing. TB: Writing—review & editing. VB: Funding acquisition, Methodology, Supervision, Writing—review & editing.

## Footnotes

^1^For example see https://yourtenderteam.co.uk/contracts/nhs-framework-agreement-for-the-supply-of-direct-oral-anticoagulants-doacs-for-the-nhs-in-england/.

^2^see https://emvo-medicines.eu/.
